# Parliament and Publications

**Published:** 1911-07-08

**Authors:** 


### PARLIAMENT AND PUBLICATIONS.

### LUNACY IN SCOTLAND.

The fifty-third annual report of the Commissioners in Lunacy for Scotland (Cd. 5,720) reveals the fact that a slight increase has taken place in the number of lunatics since the last report was published. On January 1 of the present year, exclusive of insane persons maintained at home, there were in Scotland 18,636 insane persons, including the inmates of training schools for imbecile children and of the Criminal Lunatic Department at Perth. Of these, 2,622 were maintained from private sources, 15,958 by parochial rates, and 56 at the expense of the State. The total number of lunatics in Scotland on January 1, 1910, was 18,337, so that the increase is 299. The number of male lunatics is 9,186 and of female 9,450. The number in district asylums is 10,311; the number of pauper lunatics is 15,958. During the year 1910 there were 215 private patients and 1,031 pauper patients discharged recovered; the number of private patients discharged unrecovered was 112, and the number of pauper patients 375. The proportion of patients discharged unrecovered to the average number resident has been steadily falling for the past twenty years, and last year reached its lowest point. This, as the report states, is a matter to be regretted, as, with a falling admission rate and an increased death rate, the resident population of asylums ought to have fallen rather than to have increased. This result must be largely ascribed to the lower rate of discharge of unrecovered patients who have ceased to need asylum care. The report shows that to May 1910 the pauper lunatics of Scotland maintained in District Asylums were costing the country a yearly rent per bed of £18 10s. 6d., which, added to the average cost for the food, clothing, and management of the patients at that date, gives the total cost of pauper lunatics in all District Asylums as £44 11s. 5d. per patient.

### LABELLING OF PROPRIETARY REMEDIES.

The Royal College of Physicians has recently had under consideration the question of the labelling of proprietary remedies, and this week the matter has been referred to in the House of Commons. The College is of opinion that it would be to the benefit of the public if the manufacturers and vendors of patent medicines were compelled to print on the labels the exact composition of all such medicines, and when asked by Mr. Chancellor whether he would take steps to give effect to this suggested policy the Home Secretary stated that, in his opinion, the question of the conditions of the sale of patent medicines might usefully be considered by a Select Committee next session. It may be remembered that Lord Gladstone, who preceded Mr. Churchill at the Home Office, expressed the intention of appointing a committee for a similar purpose, but Lord Gladstone went to South Africa before he had put his intention into effect. It is to be hoped that nothing will occur to prevent the institution of an inquiry into this subject at an early date, although the operation of the proposed National Insurance scheme will, in all probability, go a long way towards checking the traffic in quack remedies.

